# Overlapping research efforts in a global pandemic: a rapid systematic review of COVID-19-related individual participant data meta-analyses

**DOI:** 10.1186/s12913-023-09726-8

**Published:** 2023-07-06

**Authors:** Lauren Maxwell, Priya Shreedhar, Brooke Levis, Sayali Arvind Chavan, Shaila Akter, Mabel Carabali

**Affiliations:** 1 Heidelberger Institut Für Global Health, Universitätsklinikum Heidelberg, Im Neuenheimer Feld 130/3, 69120 Heidelberg, Germany; 2 Centre for Clinical Epidemiology, Lady Davis Institute for Medical Research, Jewish General Hospital, 3755 Cote Ste Catherine Road, Montreal, QC H3T 1E2 Canada; 3 Institute of Tropical Medicine and Public Health, Charité – Universitätsmedizin Berlin, Südring 2-3, 13353 Berlin, Germany; 4 Department of Epidemiology, Biostatistics and Occupational Health, School of Population and Global Health, McGill University, 2001 McGill College Avenue, Montréal, H3A 1G1 Canada; 5 Department of Social and Preventive Medicine, School of Public Health, Universite de Montreal, 7101 Parc Avenue, Montreal, H3N 1X9 Canada

**Keywords:** COVID-19, Individual participant data meta-analysis, Meta-analysis, Data sharing

## Abstract

**Background:**

Individual participant data meta-analyses (IPD-MAs), which involve harmonising and analysing participant-level data from related studies, provide several advantages over aggregate data meta-analyses, which pool study-level findings. IPD-MAs are especially important for building and evaluating diagnostic and prognostic models, making them an important tool for informing the research and public health responses to COVID-19.

**Methods:**

We conducted a rapid systematic review of protocols and publications from planned, ongoing, or completed COVID-19-related IPD-MAs to identify areas of overlap and maximise data request and harmonisation efforts. We searched four databases using a combination of text and MeSH terms. Two independent reviewers determined eligibility at the title-abstract and full-text stages. Data were extracted by one reviewer into a pretested data extraction form and subsequently reviewed by a second reviewer. Data were analysed using a narrative synthesis approach. A formal risk of bias assessment was not conducted.

**Results:**

We identified 31 COVID-19-related IPD-MAs, including five living IPD-MAs and ten IPD-MAs that limited their inference to published data (e.g., case reports). We found overlap in study designs, populations, exposures, and outcomes of interest. For example, 26 IPD-MAs included RCTs; 17 IPD-MAs were limited to hospitalised patients. Sixteen IPD-MAs focused on evaluating medical treatments, including six IPD-MAs for antivirals, four on antibodies, and two that evaluated convalescent plasma.

**Conclusions:**

Collaboration across related IPD-MAs can leverage limited resources and expertise by expediting the creation of cross-study participant-level data datasets, which can, in turn, fast-track evidence synthesis for the improved diagnosis and treatment of COVID-19.

**Trial registration:**

10.17605/OSF.IO/93GF2.

**Supplementary Information:**

The online version contains supplementary material available at 10.1186/s12913-023-09726-8.

## Background

The harmonisation and analysis of participant-level data and metadata for cross-study analyses, including individual participant data meta-analyses (IPD-MAs), can inform COVID-19 response through improved evaluation of diagnostic, preventative, and treatment measures. IPD-MAs have several analytic benefits over standard aggregate data meta-analyses when considering analyses of longitudinal data and the development and validation of clinical risk prediction tools [1–3]. IPD-MAs allow for joint consideration of study and subject-level heterogeneity to separate clinically relevant heterogeneity from heterogeneity related to study design or exposure and outcome ascertainment [1–3]. Separating clinically relevant from spurious heterogeneity is central to understanding whether observed differences in the risk of long COVID and COVID-19-related mortality are due to actual differences in exposure or immune response or to study-level differences in selection, ascertainment, or residual confounding.

The implementation and management of IPD-MAs are resource-intensive [1, 2, 4]. Collecting the well-characterised metadata needed to appropriately describe included studies and cleaning and harmonising participant-level data from related studies require a significant investment of time and expertise from the primary studies and the IPD-MA management team [2, 5]. Additional barriers to sharing participant-level health-related data [1], including fears of lost opportunities for publication and legal or ethical considerations, can prevent or slow down data sharing [6–8]. IPD-MAs are essential for informing research design, risk communication, and clinical practice for COVID-19. Given the significant resources needed to undertake an IPD-MA, identifying areas of overlap in exposures and outcomes of interest and inclusion criteria can foster cross-IPD-MA coordination to avoid duplication and maximise the utility of existing data.

Our research aim was to identify areas of overlap in research aims and study populations, and to identify included studies across planned, ongoing, or completed COVID-19-related IPD-MAs. We conducted a rapid systematic review to identify and describe synergies across COVID-19 IPD-MAs with a focus on study inclusion and exclusion criteria, study populations and designs, and exposure and outcomes of interest. Our working hypothesis was that there would be several areas of overlap across planned, ongoing, or completed COVID-19-related IPD-MAs. When identified early in the IPD-MA process, we expected that researchers could then exploit these cross-IPD-MA synergies to rapidly and efficiently conduct IPD-MA studies during the ongoing COVID-19 pandemic.

## Methods

We conducted a systematic search of four databases and protocol repositories, including Ovid Medline, the PROSPERO International Prospective Register of Systematic Reviews, the Open Science Foundation (OSF), and the Cochrane Database of Systematic Reviews, using a combination of MeSH (where applicable) and text terms (Additional file 1). We ran the searches on 2 June 2021, 29 October 2021, and 7 February 2022. The protocol for this systematic review was developed per the Preferred Reporting Items for Systematic Reviews and Meta-Analyses (PRISMA)-Protocol statement guidelines [9, 10]. Before implementing the searches, we uploaded the systematic review protocol and search strategies to OSF (10.17605/OSF.IO/93GF2) after unsuccessfully trying to upload the protocol to the PROSPERO Registry of Systematic Reviews, which told our team that the systematic review of IPD-MAs was not a systematic review. This systematic review is reported per the 2020 PRISMA statement (Additional file 2) [11].

### Study selection and data extraction

Eligible protocols or published studies were IPD-MAs that planned to include or included participant-level COVID-19-related health data. IPD-MAs that only included social or psychological measures and systematic reviews limited to aggregate measures rather than participant-level data from included studies were excluded. Two independent reviewers determined eligibility at the title abstract and full-text screening stages. One reviewer extracted data into a pre-piloted data extraction Google sheet. Data were subsequently reviewed by a second reviewer. Differences of opinion and discrepancies in data extraction were resolved through consensus.

### Analysis

We conducted a narrative synthesis of the results and summarise findings in a series of Sankey diagrams created in RStudio version 1.4.1103. We did not include a formal risk of bias assessment as part of this rapid systematic review, as most IPD-MAs only had a protocol available for review at the time of data extraction.

### Patient and public involvement

Patients and the public were not directly involved in this systematic review; we used publicly available data for the analysis.

## Results

**Fig.1:**
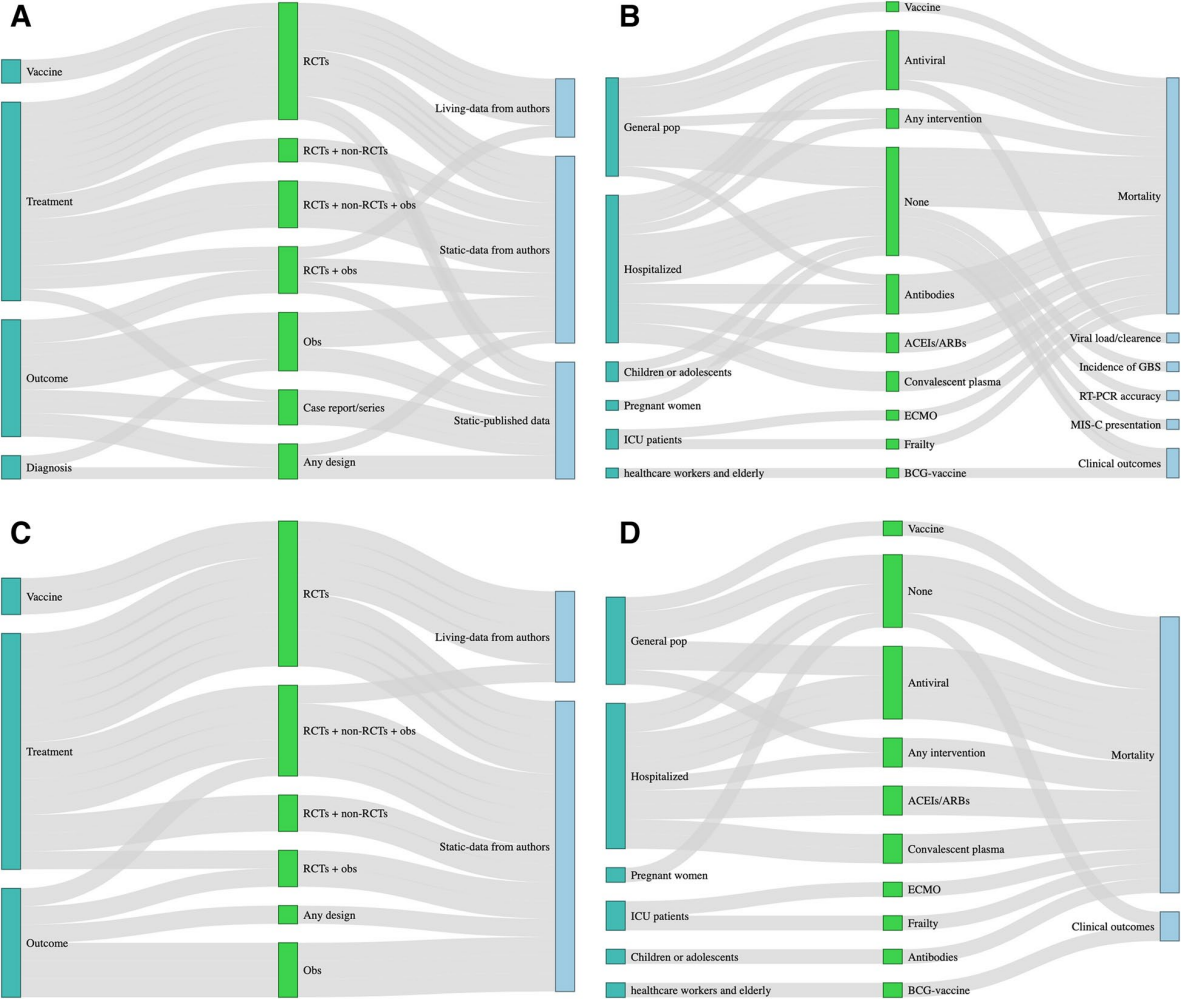
Sankey diagrams showing overlap between ongoing or completed and static or living COVID-19 IPD-MAs. **A** Shows overlap between the focus, included study designs, and type of IPD-MA for all the ongoing or completed IPD-MAs. **B** Shows overlap between the included study population, interventions/exposures, and outcomes of all the ongoing or completed IPD-MAs. **C** Shows overlap between the focus, included study designs, and type of IPD-MA for only those IPD-MAs that requested data from authors. **D** Shows overlap between the included study population, interventions/exposures, and outcomes of only those that requested data from authors. ACEIs = angiotensin-converting-enzyme inhibitors. ARBs = angiotensin II receptor blockers. BCG = Bacillus Calmette-Guérin. ECMO = extracorporeal membrane oxygenation. GBS = Guillain‐Barré syndrome. MIS-C = multisystem inflammatory syndrome in children. Obs = observational. RCTs = randomised controlled trials. RT-PCR = reverse transcription polymerase chain reaction

We reviewed 116 full texts and identified 31 COVID-19-focused health-related IPD-MAs (see Additional file 3 for the PRISMA flow diagram). The majority of IPD-MAs were identified through PROSPERO (*n* = 21), followed by Ovid Medline (*n* = 8) and OSF (*n* = 2) [12, 13]. No IPD-MAs were identified from the Cochrane Database of Systematic Reviews. The 31 ongoing or completed COVID-19 IPD-MAs are described in Table 1. As shown in the Sankey diagrams in Fig. 1A–D, there were several areas of overlap in included study populations, designs, interventions, and outcomes of interest between ongoing or completed and static or living COVID-19-related IPD-MAs. Figure 1C–D limit inference to the 21 IPD-MAs that requested data from authors, which requires more effort than IPD-MAs of data included in publications.

**Table 1:** Overview of COVID-19-focused IPD-MAs

	**First author, last name**	**Title**	**Type, status, and availability of data for IPD-MA**	**Focus**	**Population**	**Study design**	**Treatment/ exposure**	**Outcome(s)**
1	Angoulvant [14]	Initial treatment of multisystem inflammatory syndrome in children (MIS-C) and outcomes: a systematic review and meta-analysis of individual patient data; The International MIS-C Treatment Collaborative	IPD-MA; Ongoing	Pharmaceutical treatment or prophylaxis	Children or adolescents	RCTs and non-randomised intervention studies	IVIG plus glucocorticoids or glucocorticoids alone	Cardiovascular dysfunction; mortality; medical intervention rate; mechanical ventilation rate; clinical status; time-to-ICU discharge
2	Antwi-Amoabeng [15]	Clinical outcomes in COVID-19 patients treated with tocilizumab: An individual patient data systematic review	IPD-MA of published IPD only; Completed and published; Data not available	Pharmaceutical treatment or prophylaxis	Hospitalised patients	Case reports	Tocilizumab	In-hospital mortality; incidence of in-hospital complications; time-to-clinical recovery; inflammatory markers
3	Baral [16]	Individual patient data meta-analysis of renin–angiotensin–aldosterone system inhibitors in COVID-19	IPD-MA; Ongoing	Pharmaceutical treatment or prophylaxis	Hospitalised patients	RCTs and non randomised intervention studies, and longitudinal observational studies	ACEIs or ARBs	In-hospital mortality; ICU admission rate; mechanical ventilation rate; time-to-hospital discharge
4	Beyrouti [17]	Characteristics of intracerebral haemorrhage associated with COVID-19: a systematic review and pooled analysis of individual patient and aggregate data	IPD-MA of published IPD only (publication includes AD and IPD); Completed and published; Data included in the publication supplement	COVID-19 outcomes—intracerebral haemorrhage	Hospitalised patients	Any study design	N/A	Mortality, time-to-clinical recovery; discharge location
5	Campbell [18]	Predictors of COVID-19 outcomes: an individual participant meta-analysis	IPD-MA; Ongoing	COVID-19 outcomes—long COVID	General population	Any study design other than case reports	N/A	Clinical presentation of long COVID; mortality; rehospitalisation rate; time-to-hospital or ICU discharge, discharge location
6	Cao [19]	Comparative efficacy of treatments for patients infected with 2019 novel coronavirus: a systematic review and meta-analysis of individual patient data	IPD-MA; Ongoing	Pharmaceutical treatment or prophylaxis	General population	RCTs and non randomised intervention studies, and longitudinal observational studies	Lopinave/Litonawe	Mortality; time to clinical recovery
7	Cao [20]	Comparative effectiveness and safety of antiviral agents for patients with COVID-19: Protocol for a systematic review and individual patient data network meta-analysis	IPD-MA; Ongoing	Pharmaceutical treatment or prophylaxis	General population	RCTs and non randomised intervention studies, and longitudinal observational studies	Antiviral drugs alone or in any combination, including IFN-α, LPV/r, remdesivir, chloroquine, ribavirin, arbidol, and Xuebijing injection	Time to clinical recovery, all-cause mortality; vitals; mechanical ventilation rate; non-invasive ventilation rate; SAEs
8	Christophers [21]	Trends in Clinical Presentation of Children with COVID-19: A Systematic Review of Individual Participant Data	IPD-MA of published IPD only; Completed and published; Data included in the publication supplement	SARS-CoV-2 infection clinical presentation	Children or adolescents	Any study design other than case reports	N/A	Clinical presentation of COVID-19 in children
9	de Jong [12]	Clinical prediction models for mortality in COVID-19 patients: a living external validation and individual participant data meta-analysis (COVID-PRECISE)	Living IPD-MA; Ongoing	COVID-19 outcomes—mortality	Hospitalised patients	EMRs	N/A	30-day and in-hospital mortality
10	Dominguez-Rodriguez [22]	Management of mechanical circulatory support during the COVID-19 pandemic: an individual patient data meta-analysis	IPD-MA; Ongoing	Non-pharmaceutical clinical treatment	ICU patients	RCTs and longitudinal and cross-sectional observational studies	ECMO	In-hospital and ICU mortality; time-to-hospital or ICU discharge; incidence of VAP, SAEs
11	Fontes [23]	Chloroquine/hydroxychloroquine for coronavirus disease 2019 (COVID-19) – a systematic review of individual participant data	IPD-MA; Ongoing	Pharmaceutical treatment or prophylaxis	Hospitalised patients	RCTs only	Chloroquine or hydroxychloroquine	COVID-19-related mortality; All-cause mortality; ARDS incidence; hospitalisation rate; ICU admission rate, time to clinical recovery; time to viral clearance, SAEs
12	Gastine [24]	A patient-level meta-analysis on SARS-CoV-2 viral dynamics to model response to antiviral therapies	IPD-MA; Completed and published; Data uploaded to GitHub	Pharmaceutical treatment or prophylaxis	General population	RCTs and longitudinal and cross-sectional observational studies	Antiviral medication	Viral load or clearance
13	Goldfeld [25]	Prospective individual patient data meta-analysis: Evaluating convalescent plasma for COVID-19	IPD-MA; Ongoing	Non-pharmaceutical clinical treatment	Hospitalised patients	RCTs only	Convalescent plasma	Mortality; time to hospital or ICU discharge; COVID-19 severity score
14	Harwood [26]	Which children and young people are at higher risk of severe disease and death after hospitalisation with SARS-CoV-2 infection in children and young people: A systematic review and individual patient meta-analysis	IPD-MA of published IPD only; Completed and published; Data not available	COVID-19 outcomes—long COVID or mortality	Hospitalised patients	Cohorts or other longitudinal observational studies	N/A	In-hospital mortality; mechanical ventilation rate; ECMO rate; ICU admission rate
15	Hasan [27]	Guillain‐Barré syndrome associated with SARS‐CoV‐2 infection: A systematic review and individual participant data meta‐analysis	IPD-MA of published IPD only; Completed and published; Data not available	COVID-19 outcomes—multiple	General population	Case reports and case series	N/A	Incidence of GBS
16	Hong [28]	Efficacy and safety of therapeutic treatments in patients with COVID-19: a network meta-analysis	IPD-MA; Ongoing	Any treatment	General population	RCTs and non randomised intervention studies, and longitudinal observational studies	Any medical or mechanical intervention	Mortality; time-to-hospital discharge; time-to-clinical recovery; viral load; AEs and SAEs
17	Juul [29]	Interventions for treatment of COVID-19. A living systematic review with individual patient data meta-analyses, aggregate data meta-analyses, trial sequential analyses, and network meta-analysis (The LIVING Project)	Living IPD-MA; Published and ongoing; Data available from emailing authors	Any treatment	Hospitalised patients	RCTs only	Any medical or mechanical intervention	All-cause mortality; ICU admission rate; renal replacement therapy rate; mechanical ventilation rate; QoL; AEs
18	Tan [30]	Prognosis of smell and taste recovery in COVID-19 patients: a systematic review and one-stage meta-analysis of individual patient time-to-event data	IPD-MA; Ongoing	COVID-19 outcomes—neurologic	General population	Cohorts or other longitudinal observational studies	N/A	Time-to-recovery of smell or taste, time taken or extent of improvement of smell or taste
19	Korang [31]	Vaccines to prevent COVID-19: a protocol for a living systematic review with network meta-analysis including individual patient data (The LIVING VACCINE Project)	Living IPD-MA; Published and ongoing; Data not available	Vaccine efficacy	General population (limited to those who were never infected with SARS-CoV-2)	RCTs only	Any COVID-19 vaccine	All-cause mortality; COVID-19 incidence; QoL
20	Lant [32]	Neurological associations of COVID-19 (COVID-Neuro): A protocol for a systematic review and meta-analysis of individual patient data	IPD-MA; Ongoing	COVID-19 outcomes—neurologic	Hospitalised patients	RCTs and longitudinal and cross-sectional observational studies	N/A	Infection rate; in-hospital mortality; time-to-ICU discharge; time-to-hospital discharge; mechanical ventilation rate; ICU admission rate
21	Lee [33]	Cutaneous manifestations of COVID-19: a systematic review and analysis of individual patient-level data	IPD-MA of published IPD only; Completed and published; No information on data availability	COVID-19 outcomes—cutaneous	General population	Case reports and case series	N/A	Rate and spectrum of dermatological outcomes
22	Ling [34]	Interleukin-6 receptor antagonists for severe coronavirus disease 2019: a meta-analysis of individual participant data from randomised controlled trials	IPD-MA of published IPD only; Completed and published; Data not available	Non-pharmaceutical clinical treatment	Hospitalised patients	RCTs only	IL-6 inhibitors	Time-to-clinical recovery; mortality
23	Mallett [35]	At what times during infection is SARS-CoV-2 detectable and no longer detectable using RT-PCR-based tests? A systematic review of individual participant data	IPD-MA of published IPD only; Completed and published; Data available from emailing authors	SARS-CoV-2 infection diagnosis	Hospitalised patients	Case series and longitudinal studies	N/A	Timing of sample collection for accurate SARS-CoV-2 diagnosis by RT-PCR
24	Simmons [36]	Sofosbuvir/daclatasvir regimens for the treatment of COVID-19: an individual patient data meta-analysis	IPD-MA; Completed and published; No information on data availability	Pharmaceutical treatment or prophylaxis	Hospitalised patients	RCTs and non-randomised intervention studies	Sofosbuvir/daclatasvir-based regimens	Clinical recovery within 14 days of randomisation; time-to-clinical recovery; all-cause mortality; time-to-hospital discharge; composite outcome of ICU admission or requirement for invasive mechanical ventilation
25	Smith [37]	Protocol for a sequential, prospective meta-analyses [38] to rapidly address priority perinatal COVID-19 questions	Living IPD-MA; Ongoing	COVID-19 outcomes—multiple	Pregnant women	RCTs and longitudinal observational studies	N/A	Adverse birth outcomes; pregnancy-related mortality and morbidity; vertical transmission rate of COVID-19
26	Speich [13]	Efficacy and safety of remdesivir in hospitalised patients with COVID-19: Systematic review and individual patient data meta-analysis of randomised trials	IPD-MA; Ongoing	Pharmaceutical treatment or prophylaxis	Hospitalised patients	RCTs only	Remdesivir	28- and 60-day mortality; mechanical ventilation rate; duration of mechanical ventilation; clinical status; time-to-clinical recovery; time-to-ICU or hospital discharge; QoL; Viral load or clearance; AEs or SAEs
27	Subramaniam [39]	Characteristics and Outcomes of Patients with Frailty Admitted to ICU with Coronavirus Disease 2019: An Individual Patient Data Meta-Analysis	IPD-MA; Completed and published; No information on data availability	COVID-19 outcomes—multiple	ICU patients	Cohorts or other longitudinal observational study only	Frailty	In-hospital mortality; time-to-hospital or ICU discharge; mechanical ventilation rate; non-invasive ventilation rate; ECMO rate; renal replacement therapy rate, vasoactive infusion rate; ICU bed occupancy; discharge location
28	Tasoudis[40]	Survival analysis of IL-6 inhibitors versus standard of care for COVID-19: a meta-analysis of individual patient data from randomised trials	IPD-MA of published IPD only; Completed and published; No information on data availability	Pharmaceutical treatment or prophylaxis	General population	RCTs only	IL-6 inhibitors	Mortality; ICU admission rate; hospital discharge rate; mechanical ventilation rate
29	Troxel [41]	Association of Convalescent Plasma Treatment With Clinical Status in Patients Hospitalised With COVID-19: A Meta-analysis	Living IPD-MA; Published and ongoing; No information on data availability	Non-pharmaceutical clinical treatment	Hospitalised patients	RCTs only	Convalescent plasma	14- and 28-day mortality; COVID-19 severity score
30	van Werkhoven [42]	Anytime Live and Leading Interim* meta-analysis of the impact of BCG vaccine in health care workers and elderly during the SARS-CoV-2 pandemic (ALL-IN-META-BCG-CORONA)	Living IPD-MA; Ongoing	Vaccine efficacy	Health care workers; elderly	RCTs only	BCG-vaccine	COVID-19 incidence; hospitalisation rate; infection rate; time to clinical recovery; time to hospital discharge
31	Victory [38]	ACEi/ARB medications for hospitalised patients with COVID-19: an individual patient data (IPD)-based pooled analysis	IPD-MA; Ongoing	Pharmaceutical treatment or prophylaxis	Hospitalised patients	RCTs only	ACEIs or ARBs	COVID-19 severity score; time to hospital discharge; duration of mechanical ventilation; 14-day mortality; 30-day mortality, AEs or SAEs

*ACEIs* Angiotensin-converting-enzyme inhibitors, *AD* Aggregate data, *AE* Adverse event, *ARBs* Angiotensin II receptor blockers, *ARDS* Acute respiratory distress syndrome, *BCG* Bacillus Calmette-Guérin, *ECMO* Extracorporeal membrane oxygenation. *EMRs* Electronic medical records, *GBS* Guillain‐Barré syndrome, *ICU* Intensive care unit, *IL-6* Interleukin 6, *IPD* Individual participant data, *IPD-MA* Individual participant data meta-analysis, *IVIG* Intravenous immunoglobulins, *MIS-C* Multisystem inflammatory syndrome in children, *N/A* Not applicable, *QoL* Quality of life, *RCT* Randomized controlled trial, *RT-PCR* Reverse transcription polymerase chain reaction, *SAE* Serious adverse event, *SARS-CoV-2* Severe acute respiratory syndrome coronavirus, *VAP* Ventilator-associated pneumonia

### Study designs

Ten IPD-MAs included randomised controlled trials (RCTs), non-randomised intervention studies, or longitudinal observational studies; an additional 10 IPD-MAs were limited to RCTs only. Three IPD-MAs included RCTs and longitudinal or cross-sectional observational studies [22, 24, 32]. Two IPD-MAs had case reports and case series [27, 33]. One IPD-MA each was limited to case reports [15], medical records [12], and case series and longitudinal studies [35]. One IPD-MA included any study design [17]; two others included any study design other than case reports [18, 21].

### Populations

More than half of the 31 IPD-MAs were conducted with data from hospitalised or intensive care unit (ICU) patients (*n* = 17). Ten IPD-MAs included data from the general population, and two IPD-MAs were limited to children or adolescents [14, 21]. One IPD-MA was conducted with pregnant women [37] and one with older adults and health care workers [42]. Most IPD-MAs were not limited by geography (*n* = 28). One IPD-MA was limited to studies in the US and Canada [38], another to the US, Europe, and China [29], and one to China [19].

### Treatment or exposure

Sixteen IPD-MAs focused on the evaluation of medical treatments, including antivirals (*n* = 6) [13, 19, 20, 23, 24, 36], antibodies (*n* = 4) [14, 15, 34, 40], angiotensin-converting-enzyme inhibitors (ACEIs) or angiotensin II receptor blockers (ARBs; *n* = 2) [16, 38], convalescent plasma (*n* = 2) [25, 41], COVID-19 vaccines (*n* = 1) [31], and the Bacillus Calmette–Guérin (BCG)-vaccine (*n* = 1) [42]. One IPD-MA focused on extracorporeal membrane oxygenation (ECMO) [22]. Two IPD-MAs evaluated any medical or mechanical intervention, including ECMO [28, 29]. One IPD-MA had frailty as the exposure [39].

### Outcomes

IPD-MAs shared a number of common outcomes, including rate of mechanical (*n* = 8) or non-invasive ventilation (*n* = 2) [20, 39], ECMO rate (*n* = 2) [26, 39], rate of serious adverse events (SAEs) or adverse events (AEs; *n* = 7), viral clearance or viral load (*n* = 4) [13, 23, 24, 28], COVID-19 infection rate (*n* = 3) [31, 32, 42], rate of hospitalization or rehospitalization (*n* = 3) [18, 23, 42] or admittance to the ICU (*n* = 6), time-to-hospital or ICU discharge (*n* = 12), hospital discharge location (*n* = 3) [17, 18, 39], time-to-clinical recovery (*n* = 10), COVID-19 severity score (*n* = 3) [25, 38, 41], quality of life-related measures (*n* = 3) [13, 29, 31], and mortality (*n* = 24). Areas of overlap in mortality measures included IPD-MAs that assessed in-hospital mortality (*n* = 7) and all-cause mortality (*n* = 5). One IPD-MA assessed ICU mortality [22], and another pregnancy-related mortality [37]. IPD-MAs that specified time-to-death, included: 14-day mortality (*n* = 2) [38, 41], 28-day mortality (*n* = 2) [13, 41], 30-day mortality (*n* = 2) [12, 38], and 60-day mortality (*n* = 1) [13]. Individual IPD-MAs focused on the clinical presentation of long COVID-19 [18] and COVID-19 in children [21]; time-to-recovery of smell or taste [30]; incidence of Guillain–Barre syndrome [27]; rate and spectrum of dermatological outcomes in COVID-19 patients [33]; viral load at of sample collection and its effect on the accuracy of RT-PCR [35]; and adverse birth outcomes and vertical transmission in pregnant women with COVID-19 [37].

### Types of IPD-MAs

Ten IPD-MAs were limited to published IPD, which means that the group conducting the IPD-MA did not contact authors to request data. Five were living IPD-MAs where datasets and related findings are regularly updated as evidence becomes available [29, 31, 37, 41, 42]. Living IPD-MAs included a real-time IPD-MA [41], a network IPD-MA [31], and an IPD-MA of IPD-MAs [29]. Living IPD-MAs focused on COVID-19 vaccines [31], BCG vaccine [42], any treatment [29], convalescent plasma [41], and issues of interest to perinatal populations [37], Four of the five living IPD-MAs were limited to RCTs [29, 31, 41, 42].

### Availability of data from IPD-MAs

Fifteen IPD-MAs were published when we submitted the manuscript for publication. Three published IPD-MAs made their data available through GitHub (*n* = 1) [24] or the journal supplement (*n* = 2) [17, 21]. Two published IPD-MAs stated that interested researchers could request the dataset from the study team [29, 35], and five said that data would not be made available [15, 26, 27, 31, 34]. Five others did not include a statement related to data availability [33, 36, 39–41]. Three of the living IPD-MAs were published [29, 31, 41], although only one indicated that data could be requested from the study team [29].

## Discussion

IPD-MAs are an essential tool for the rapid evidence generation needed to inform clinical practice, making them a vital part of the research response to emerging pathogens [43]. We conducted a rapid systematic review to identify ongoing or completed COVID-19-related IPD-MAs. There were many areas of overlap in the 31 COVID-19-related IPD-MAs, including in study design and population, exposure, and outcomes of interest. In particular, the 14 IPD-MAs that evaluated the same medical exposures (antivirals, antibodies, ACEIs and ARBs, and convalescent plasma) represent a missed opportunity to exploit synergies. Most IPD-MA protocols were registered on PROSPERO, which could flag these areas of overlap when researchers submit their protocol. IPD-MAs require a significant investment of time and expertise, both from the team conducting the IPD-MA and the groups contributing data to the IPD-MA. Rapidly identifying and exploiting shared inclusion criteria can help facilitate evidence generation and avoid unnecessary duplication of effort.

We identified at least 10 IPD-MAs that limited their analysis to data included in published reports. While IPD-MAs that are limited to published IPD have been conducted previously, the volume of the research response to COVID-19 coupled with the push for reproducibility and transparency have likely facilitated the rise in IPD-MAs of data that were included in the study publications. Almost half of the IPD-MAs of published data included case study or case series data (*n* = 4/10; 40%) [15, 27, 33, 35]. Given that the utility of the IPD-MA is limited by the quality of the studies that contribute data [2], findings from these rapidly produced IPD-MAs should be considered preliminary and updated when more detailed and less selective participant-level datasets become available. This finding is in keeping with a methodological review of published data that compared the methodological and reporting quality of COVID-19 and non-pandemic research and found a reduction in quality in the former [44].

While we reviewed the protocols for all IPD-MAs, we could only identify the restriction to published IPD for those IPD-MAs that had published their analyses, which suggests a need to clarify inclusion criteria in IPD-MA protocols to specify the intent to limit inference to published IPD. Some of the unpublished studies identified in our review may be misclassified as having the classical approach to conducting an IPD-MA, which includes the challenges associated with requesting the data from the data producers.

Living IPD-MAs are regularly updated as more evidence becomes available, representing substantial investments. There was overlap in study design, exposure, and outcome measurements in several of the five living IPD-MAs and between the living IPD-MAs and static IPD-MAs, which represents an opportunity to share limited resources and expedite findings.

Only a few IPD-MAs of data received from authors had been published when this manuscript was submitted for publication (*n* = 5/21; 24%), so we could not quantify the overlap in datasets across IPD-MAs that collected datasets from research teams which would be an important measure of cross-IPD-MA redundancy in efforts. Only three of the ten published IPD-MAs had made data available through a repository or the publication of supplementary materials [17, 21, 24], which suggests a continued need to encourage data sharing.

Working collaboratively to harmonise and share data across related IPD-MAs would maximise limited resources and shorten the timeline to deliver results that best inform clinical and public health practice. Testing the same hypotheses, especially with the same study designs or populations, represents a missed opportunity to evaluate novel hypotheses. Our findings support similar calls from a living review of COVID-19-related clinical trials and a scoping review of COVID-19-related data sharing platforms, which urged coordination across initiatives to reduce redundancies [45]. We propose the creation of a task force to identify concrete steps to enable cross-initiative collaboration and ensure that the harmonised participant-level data and study-related metadata correspond to the findable, accessible, interoperable, reusable (FAIR) principles for data resources [46]. These steps could include a cross-platform algorithm that uses natural language processing to alert researchers to similar initiatives during protocol deposition. The pandemic's global scope and rapidly evolving nature underscore the need for more meta-collaborations to bring together data-sharing efforts and cross-national analyses. The coordination of ongoing or planned IPD-MAs is a good starting place.

## Conclusions

IPD-MAs are important for informed research and public health response to COVID-19. To identify areas of overlap, we conducted a rapid systematic review of completed or ongoing COVID-19 IPD-MAs. We identified 31 COVID-19-related IPD-MAs, including five living IPD-MAs, and found several areas of overlap in study designs, populations, exposures, and outcomes of interest. This review shows several potential areas of collaboration across related IPD-MAs which can leverage limited resources and expertise by expediting the creation of cross-study participant-level datasets. This, in turn, can fast-track evidence synthesis for the improved diagnosis and treatment of COVID-19.

### Supplementary Information

**Additional file 1. **Database-specific search strategies.

**Additional file 2. **PRISMA 2020 Checklist.

**Additional file 3. **PRISMA Flow Diagram.

## Data Availability

The research protocol is available at the Open Science Foundation, registration number: 10.17605/OSF.IO/93GF2. A spreadsheet with comprehensive information on all planned or concluded IPD-MAs described in this review is available on Zenodo (10.5281/zenodo.6623480) under the Creative Commons Attribution 4.0 International (CC BY 4.0) license.

## Abbreviations

ACEIs: Angiotensin-converting-enzyme inhibitors
AD: Aggregate data
AEs: Adverse events
ARBs: Angiotensin II receptor blockers
ARDS: Acute respiratory distress syndrome
BCG: Bacillus Calmette–Guérin
ECMO: Extracorporeal membrane oxygenation
EMRs: Electronic medical records
FAIR: Findable, accessible, interoperable, reusable
GBS: Guillain‐Barré syndrome
ICU: Intensive care unit
IL-6: Interleukin 6
IPD: Individual participant data
IPD-MAs: Individual participant data meta-analyses
IVIG: Intravenous immunoglobulins
MeSH: Medical Subject Headings
MIS-C: Multisystem inflammatory syndrome in children
Obs: Observational
OSF: Open Science Foundation
PRECISE: Precise Risk Estimation to optimise COVID-19 Care for Infected or Suspected patients in diverse sEttings
PRISMA: Preferred Reporting Items for Systematic Reviews and Meta-Analyses
PROSPERO: International Prospective Register of Systematic Reviews
QoL: Quality of life
RCTs: Randomised controlled trials
RT-PCR: Reverse transcription polymerase chain reaction
SAEs: Serious adverse events
SARS-CoV-2: Severe acute respiratory syndrome coronavirus
VAP: Ventilator-associated pneumonia
