# Supplementary material for: Overlapping research efforts in a global pandemic: a rapid systematic review of COVID-19-related individual participant data meta-analyses
Source: BMC Health Serv Res. 2023 Jul 6;23:735. doi: 10.1186/s12913-023-09726-8 (PMC10327330; doi:10.1186/s12913-023-09726-8)
Supplement: Supplementary file 1 — Additional file 1. Database-specific search strategies. [file 12913_2023_9726_MOESM1_ESM.docx]

**Overlapping research efforts in a global pandemic. Results from a rapid systematic review of COVID-19-related individual participant data meta-analyses.**

**Supplementary Information**

# Additional File 1. Search strategy for each database

| **Ovid(Medline)(R)** | |
| --- | --- |
| 1 | ((individual adj 1 (patient or participant or subject or data)) or ((patient or participant or subject) adj1 level) or IPD-MA or IPD or pooled data).m_titl. |
| 2 | Coronavirus Infections/ or Coronavirus/ or SARS-CoV-2/ or COVID-19/ |
| 3 | ("2019 nCoV" or 2019nCoV or coronavir* or coronovir* or COVID or COVID19 or HCoV* or "nCov 2019" or "SARS CoV2" or "SARS CoV 2" or SARSCoV2 or "SARSCoV 2" OR ((corona* or corono*) adj1 (virus* or viral* or virinae*))).m_titl. |
| 4 | 2 OR 3 |
| 5 | 1 AND 4 |
| **PROSPERO International prospective register of systematic reviews**  (https://www.crd.york.ac.uk/prospero/#searchadvanced) | |
| (((coronavirus or corona-virus) AND (wuhan or beijing or shanghai or Italy or South-Korea or korea or China or Chinese or 2019-nCoV or nCoV or COVID-19 or Covid19 or SARS-CoV* or SARSCov2 or ncov)) OR (pneumonia AND Wuhan) or "COVID-19" or "2019-nCoV" or "SARS-CoV" or SARSCOV2 or 2019-nCov or "2019 coronavirus" or "2019 corona virus" or covid19 or ncov OR "novel corona virus" or "new corona virus" or "nouveau corona virus" or "2019 corona virus" OR "novel coronavirus" or "new coronavirus" or "nouveau coronavirus" or "2019 coronavirus") AND (IPD):RT NOT Animal:DB | |
| **Cochrane Database of Systematic Reviews**  (https://www.cochranelibrary.com/cdsr/reviews) | |
| Cochrane Reviews: Topic: Infectious Disease - COVID-19  Cochrane Protocols: Topic: Infectious Disease - COVID-19 | |
| **Open Science Foundation**  (https://osf.io/) | |
| (COVID-19 OR SARS-CoV-2 OR COVID OR Corona*) AND ("individual participant" OR "individual patient" OR "participant level" OR "patient level") AND (Registrations OR Projects) | |
