# Supplementary material for: Overlapping research efforts in a global pandemic: a rapid systematic review of COVID-19-related individual participant data meta-analyses
Source: BMC Health Serv Res. 2023 Jul 6;23:735. doi: 10.1186/s12913-023-09726-8 (PMC10327330; doi:10.1186/s12913-023-09726-8)
Supplement: Supplementary file 3 — Additional file 3. PRISMA Flow Diagram. [file 12913_2023_9726_MOESM3_ESM.docx]

**Overlapping research efforts in a global pandemic. Results from a rapid systematic review of COVID-19-related individual participant data meta-analyses.**

**Supplementary Information**

**Additional File 3. PRISMA Flow Diagram***

Studies included in review

(n = 31)

**Screening**

**Included**

Reports assessed for eligibility

Search date: 2 Jun 2021 (n = 53)

Search date: 28 Oct 2021 (n = 29)

Search date: 8 Feb 2022 (n = 34)

Reports excluded:

Not COVID-19 related (n = 8)

COVID-19 related, but not an IPD-MA (n = 75)

Duplicate (n = 2)

Reports sought for retrieval

Search date: 2 Jun 2021 (n = 53)

Search date: 28 Oct 2021 (n = 29)

Search date: 8 Feb 2022 (n = 34)

Reports not retrieved

(n = 0)

Records screened

Search date: 2 Jun 2021 (n = 2844)

Search date: 28 Oct 2021 (n = 160)

Search date: 8 Feb 2022 (n = 217)

Records excluded

Search date: 2 Jun 2021 (n = 2791)

Search date: 28 Oct 2021 (n = 131)

Search date: 8 Feb 2022 (n = 183)

**Identification of studies via databases and registers**

Records identified from:

Search date: 2 Jun 2021

Cochrane DSR (n = 2748)

PROSPERO (n = 93)

OSF (n = 3)

Search date: 28 Oct 2021

Ovid(Medline) (n = 160)

Search date: 9 Feb 2022

Ovid(Medline) (n = 22)

Cochrane Database of Systematic Reviews (n = 26)

Cochrane Database of Protocols (n = 9)

PROSPERO (n = 93)

OSF Registrations (n=103)

Records removed *before screening*:

Search date: 2 Jun 2021

Duplicate records removed (n = 0)

Search date: 28 Oct 2021

Duplicate records removed (n = 0)

Search date: 9 Feb 2022

Duplicate records removed (n = 36)

**Identification**

IPD-MA=individual participant data meta-analysis. OSF=Open Science Foundation.

**From:*  Page MJ, McKenzie JE, Bossuyt PM, Boutron I, Hoffmann TC, Mulrow CD, et al. The PRISMA 2020 statement: an updated guideline for reporting systematic reviews. BMJ 2021;372:n71. doi: 10.1136/bmj.n7
